# Targeting the transcription factor receptor LXR to treat clear cell renal cell carcinoma: agonist or inverse agonist?

**DOI:** 10.1038/s41419-019-1654-6

**Published:** 2019-05-28

**Authors:** Guangzhen Wu, Qinglian Wang, Yingkun Xu, Jianyi Li, Hongge Zhang, Guanghui Qi, Qinghua Xia

**Affiliations:** 1 Department of Urology, Shandong Province Hospital Affiliated to Shandong University, Jinan, China; 2 Department of Urology, The First Affiliated Hospital of Dalian Medical University, Dalian, China; 3 Department of Nephrology, Shandong Province Hospital Affiliated to Shandong University, Jinan, China; 4 Department of Urology, Tengzhou Hospital of Traditional Chinese Medicine, Tengzhou, China; 5 Department of Urology, The First Hospital of Zibo City, Zibo, China

**Keywords:** Cancer metabolism, Renal cell carcinoma

## Abstract

Growing evidence indicates that clear cell renal cell carcinoma (ccRCC) is a metabolism-related disease. Changes in fatty acid (FA) and cholesterol metabolism play important roles in ccRCC development. As a nuclear transcription factor receptor, Liver X receptor (LXR) regulates a variety of key molecules associated with FA synthesis and cholesterol transport. Therefore, targeting LXR may provide new therapeutic targets for ccRCC. However, the potential regulatory effect and molecular mechanisms of LXR in ccRCC remain unknown. In the present study, we found that both an LXR agonist and an XLR inverse agonist could inhibit proliferation and colony formation and induce apoptosis in ccRCC cells. We observed that the LXR agonist LXR623 downregulated the expression of the low-density lipoprotein receptor (LDLR) and upregulated the expression of ABCA1, which resulted in reduced intracellular cholesterol and apoptosis. The LXR inverse agonist SR9243 downregulated the FA synthesis proteins sterol regulatory element-binding protein 1c (SREBP-1c), fatty acid synthase (FASN) and stearoyl-coA desaturase 1 (SCD1), causing a decrease in intracellular FA content and inducing apoptosis in ccRCC cells. SR9243 and LXR623 induced apoptosis in ccRCC cells but had no killing effect on normal renal tubular epithelial HK2 cells. We also found that SRB1-mediated high-density lipoprotein (HDL) in cholesterol influx is the cause of high cholesterol in ccRCC cells. In conclusion, our data suggest that an LXR inverse agonist and LXR agonist decrease the intracellular FA and cholesterol contents in ccRCC to inhibit tumour cells but do not have cytotoxic effects on non-malignant cells. Thus, LXR may be a safe therapeutic target for treating ccRCC patients.

## Introduction

Renal cell carcinoma (RCC) is one of the most common malignant tumours in humans. In 2017, there were 63,900 new cases of RCC and 14,400 deaths from RCC in the United States^1^. ccRCC is the most common histological subtype of RCC, accounting for 75–80% of RCC cases^2^. Surgery is the main treatment approach, and surgical removal of localised ccRCC usually leads to improved long-term disease-free survival (DFS)^3^. However, ~20 to 30% of ccRCC patients develop metastatic renal cell carcinoma (mRCC) after diagnosis. In addition, 30% of patients with newly diagnosed local disease have metastasis^4^. Unfortunately, clinical outcomes after treatment with agents such as tyrosine kinase inhibitors (TKIs) and mammalian target of rapamycin (mTOR) inhibitors have not shown satisfactory improvement due to tumour recurrence and metastasis^5^. Therefore, understanding the underlying molecular mechanisms of ccRCC and identifying new therapeutic strategies are important.

Non-malignant cells generally support their metabolism via oxidative phosphorylation through the tricarboxylic acid (TCA) cycle, whereas tumour cells utilise aerobic glycolysis, which is known as the Warburg effect. Excess glycolytic metabolites produced by the Warburg effect are integrated into lipid production and other metabolic pathways in tumour cells, such as the de novo synthesis of FAs, nucleotide production and amino acid synthesis, which are essential for the rapid growth of cancer cells. Recent studies have found that ccRCC has a more pronounced Warburg effect than other tumours (glioma, lung cancer)^6^. Therefore, targeting LXR could cause a decrease in the downstream genes associated with the Warburg effect, such as FA synthesis genes, and thereby have an inhibitory effect in ccRCC. Another difference between cancer cells and non-malignant cells is that cancer cells exhibit high expression of lipogenic enzymes, whereas non-malignant cells primarily acquire lipids from exogenous sources^7^. FAs are synthesised by the rate-limiting enzymes FASN and SCD1. As important structural components of the cell membrane, FAs play a vital role in tumour development^8^. Increased expression of FASN, SCD1 and SREBP-1c is associated with multiple forms of cancer, and lipogenesis inhibitors that block the activities of FASN^9^, SCD1 and SREBP-1c have been shown to reduce cancer cell proliferation and induce apoptosis^10^.

A growing number of studies have shown that ccRCC is a metabolic disease^11^ and that the total cholesterol (TC) and cholesterol ester (CE) contents in ccRCC tissues are higher than those in normal kidney tissues^12^. Changes in intracellular cholesterol have profound effects on cell function, including signal transduction, membrane plasticity, and membrane migration^13^. Cholesterol can be synthesised via de novo synthesis under the action of the important rate-limiting enzyme HMGCR. Low-density lipoprotein receptor (LDLR) is mainly involved in cholesterol influx, whereas ATP binding cassette subfamily A member 1 (ABCA1) is involved in cholesterol efflux. The body maintains a balance of cellular cholesterol levels in a variety of ways^14^, and a cholesterol imbalance can lead to diseases such as atherosclerosis and tumours^15,16^. Usually, the cellular cholesterol content is regulated by the balance among cholesterol synthesis, uptake and efflux. In cancer, these homoeostatic processes are often disrupted to promote cell survival and uncontrolled growth^17^.

LXR is an important transcription factor receptor in the nucleus and consists of two subtypes: LXRα and LXRβ. LXRα and LXRβ have extensive sequence homology but no obvious tissue distribution similarities. LXRα is highly expressed in the liver, intestine, adipose tissue and macrophages, whereas LXRβ is ubiquitously expressed^18^. LXRs form specific heterodimers with retinol X receptor alpha (RXRα) and bind to specific DNA recognition sequences, termed LXR response elements (LXRes). In the absence of ligand, the LXR–RXR complex binds to a co-repressor, such as SMRT or NCOR, and inhibits the expression of target genes, such as the FA synthesis genes SREBP-1c, FASN, and SCD1 and the cholesterol transport-related genes IDOL and ABCA1. Ligands that bind to LXRs cause conformational changes in the LXR–RXR complex, and these changes lead to release of the co-repressor and the addition of synergistic activators, such as histone acetyltransferases, thereby increasing target gene transcription^19,20^. Therefore, LXRs plays crucial roles in regulating the metabolism of FAs and cholesterol. LXR agonists can reduce intracellular cholesterol content, and LXR inverse agonists can reduce intracellular FA content. As FAs and cholesterol are cell membrane structures, we speculate that LXR is stably expressed in tumour cells to maintain tumour cell proliferation but that LXR agonists or inverse agonists can disrupt the balance, causing tumour cell death. A recent study showed that ccRCC rarely relies on the TCA cycle after glucose intake and most often utilises aerobic glycolysis through the Warburg effect, and the Warburg effect produces a large number of metabolic intermediates, such as acetyl-CoA, which are synthetic precursors of FAs and cholesterol^21^. Therefore, intervening in FA synthesis pathways may have antitumour effects in ccRCC while having no cytotoxic effects on normal cells. Flaveny found that an LXR inverse agonist, SR9243, could induce LXR and co-repressor interactions. SR9243 selectively targets the Warburg effect and a variety of proteins that inhibit FA synthesis, such as SREBP-1c, FASN and SCD1, and inhibits FA production^8^. While LXR623 is a synthetic LXR agonist, recent studies have demonstrated that LXR623 reduces intracellular cholesterol content in gliomas, thereby inhibiting tumour growth and promoting tumour cell death^22^. In our study, we found that both an LXR agonist and inverse agonist induce apoptosis in ccRCC cells. We observed that LXR623 downregulated the expression of LDLR while upregulating ABCA1, which resulted in a reduction in intracellular cholesterol content and the occurrence of apoptosis. While SR9243 downregulated the FA synthesis proteins SREBP-1c, FASN and SCD1, causing a decrease in intracellular FA content and inducing apoptosis in ccRCC cells. SR9243 and LXR623 induced apoptosis in ccRCC cells but did not have cytotoxic effects on HK2 cells. Therefore, LXR may be a safe potential therapeutic target for treating ccRCC patients.

## Results

### The LXR agonist LXR623 and LXR inverse agonist SR9243 killed ccRCC cells in a concentration- and time-dependent manner

**Fig. 1:**
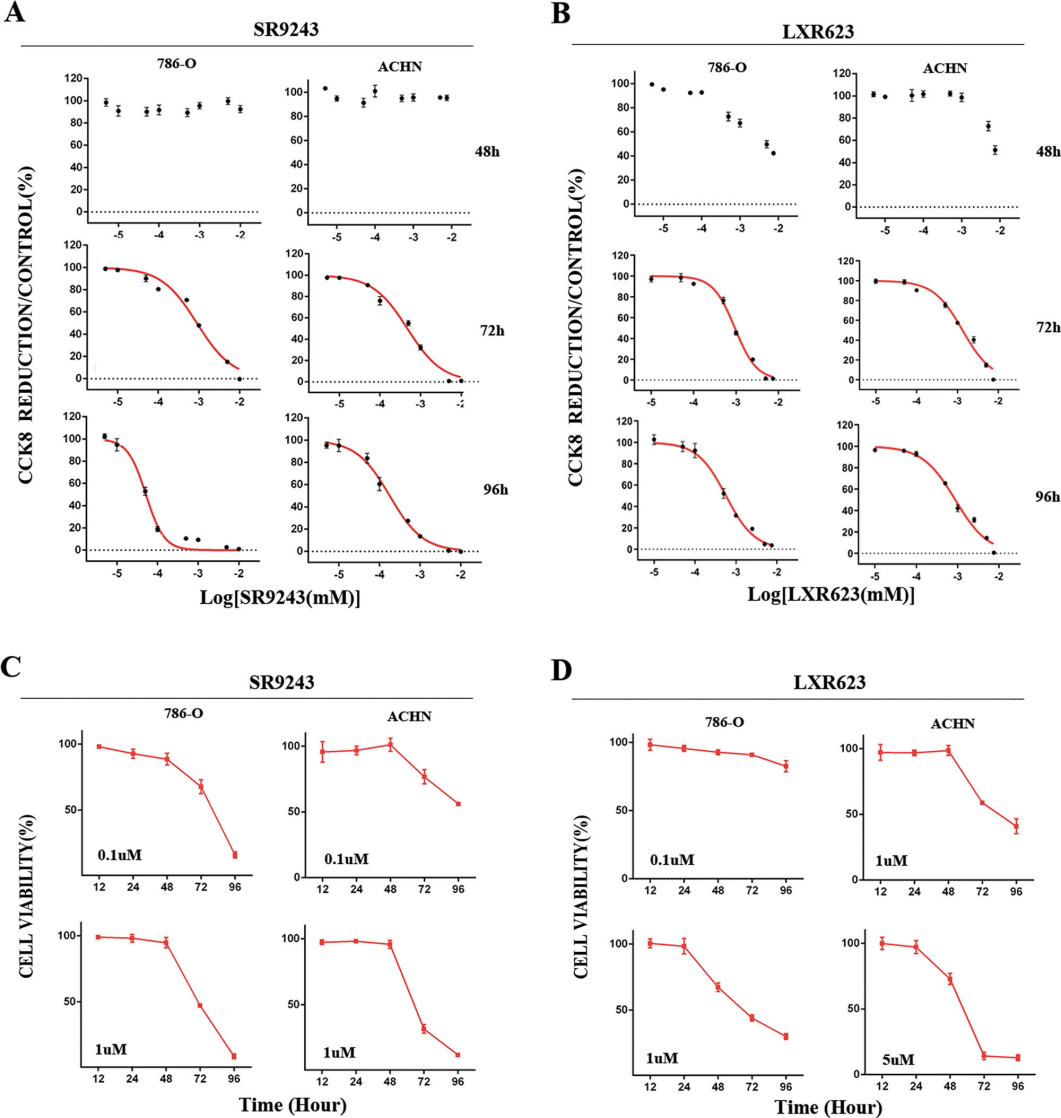
Killing effects of LXR623 and SR9243 on ccRCC cells. **a** CCK8 assays were used to detect the difference in viability of ccRCC cells (786-O and ACHN) after 48, 72 and 96 h of treatment with different doses of SR9243. **b** CCK8 assays were used to detect the difference in viability of renal cancer cells (786-O and ACHN) after 48, 72 and 96 h of treatment with different doses of LXR623. **c** CCK8 assays were used to detect cell viability at different times using the same dose use of SR9243 (0.1 µM or 1 µM). **d** CCK8 assays were used to detect cell viability at different times using the same dose of LXR623 (0.1 µM, 1 µM or 5 µM)

To investigate whether SR9243 and LXR623 have killing effects on ccRCC cells, we performed CCK8 experiments with ACHN and 786-O cell lines. We applied the agonists at different concentrations. We found that SR9243 effectively killed cancer cells at nanomolar concentrations (786-O cells [IC50]: 51.6 nM, ACHN cells [IC50]: 178.2 nM). Unexpectedly, we found that LXR623 also effectively killed cancer cells at nanomolar concentrations (786-O cells [IC50]: 569.3 nM, ACHN cells [IC50]: 874.2 nM) (Fig. 1a, b). To investigate whether the two drugs killed ccRCC cells in a time-dependent manner, we performed CCK8 experiments at the same drug concentration for different lengths of time. We found that the killing effect of each drug increased in strength with increasing drug action time (Fig. 1c, d). The findings indicated that SR9243 and LXR623 kill ccRCC cells in concentration-dependent and time-dependent manners.

### SR9243 and LXR623 inhibit cell proliferation and induce apoptosis of ccRCC cells via the endogenous apoptotic pathway in vitro

**Fig. 2:**
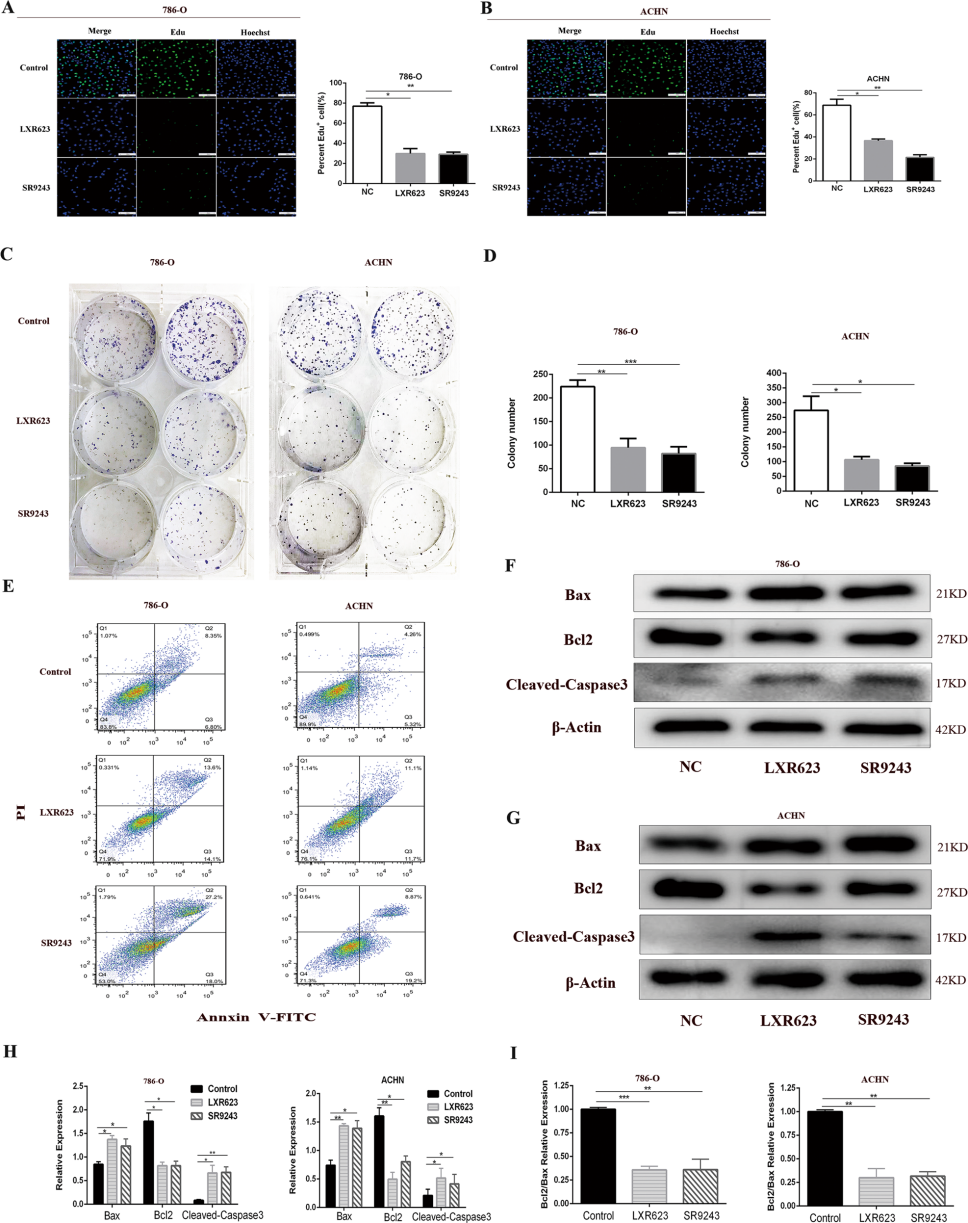
LXR623 and SR923 inhibit ccRCC cell proliferation and promote apoptosis in vitro. **a**, **b** EdU assay: cell staining using EdU (green) and the nuclear dye Hoechst (blue); 7860-O cells treated with 0.1 µM SR9243 or 1 µm LXR623 for 72 h, ACHN cells treated with 0.1 µM SR9243 or 5 µm LXR623 for 72 h. Scale bar: 100 μm. **c**, **d** Colony formation assays: SR9243 (0.1 µM) and LXR623 (0.1 µM and 1 µM) were applied to 786-O and ACHN cells for 72 h. **e** 786-O and ACHN cells were stained with Annexin-V FITC/PI and analysed via flow cytometry. SR9243 (0.1 µM) and LXR623 (1 µM for 786-O cells, 5 µM for ACHN cells) acted on the two cell lines. **f**–**i** Western blotting experiments were performed by treating three groups of cells with DMSO, 0.1 µM SR9243 or LXR623 (786-O, 0.1 µM; ACHN, 1 µM) for 48 h and then extracting proteins. The protein levels of Bax, Bcl2, and Cleaved-Caspase3 were determined via immunoblotting. All data are expressed as the mean ± S.E.M. The experiment was repeated at least three times. Statistical significance was determined using two-tailed Student’s *t-*test or one-way ANOVA. *p < 0.05; *p < 0.01

To investigate whether SR9243 and LXR623 inhibit ccRCC cell proliferation, we performed EdU cell proliferation assays and colony formation assays. As shown by the EdU analysis, SR9243 and LXR623 greatly reduced the numbers of EdU-positive ACHN and 786-O cells, suggesting that SR9243 and LXR623 can effectively inhibit cell proliferation (Fig. 2a, b). We performed colony formation assays in the two cell lines at defined time intervals. We observed that after SR9243 and LXR623 treatment, the colony formation ability of cells was significantly impaired (Fig. 2c, d). We then demonstrated via flow cytometry that both drugs significantly induced apoptosis in the two cell lines (Fig. 2e). We also examined the expression of the apoptosis-related proteins Bax, Bcl2 and Cleaved-Caspase3. Interestingly, both SR9243 and LXR623 upregulated the apoptotic protein Bax and downregulated the apoptosis-inhibiting protein Bcl2, and the expression of the apoptosis-executing protein Cleaved-Caspase3 was upregulated by both drugs (Fig. 2f–h). The Bcl2/Bax ratio was significantly decreased following drug treatment (Fig. 2i), which demonstrated that SR9243 and LXR623 induce apoptosis in ccRCC cells in vitro through an endogenous apoptotic pathway.

### SR9243 and LXR623 kill ccRCC cells through different mechanisms

**Fig. 3:**
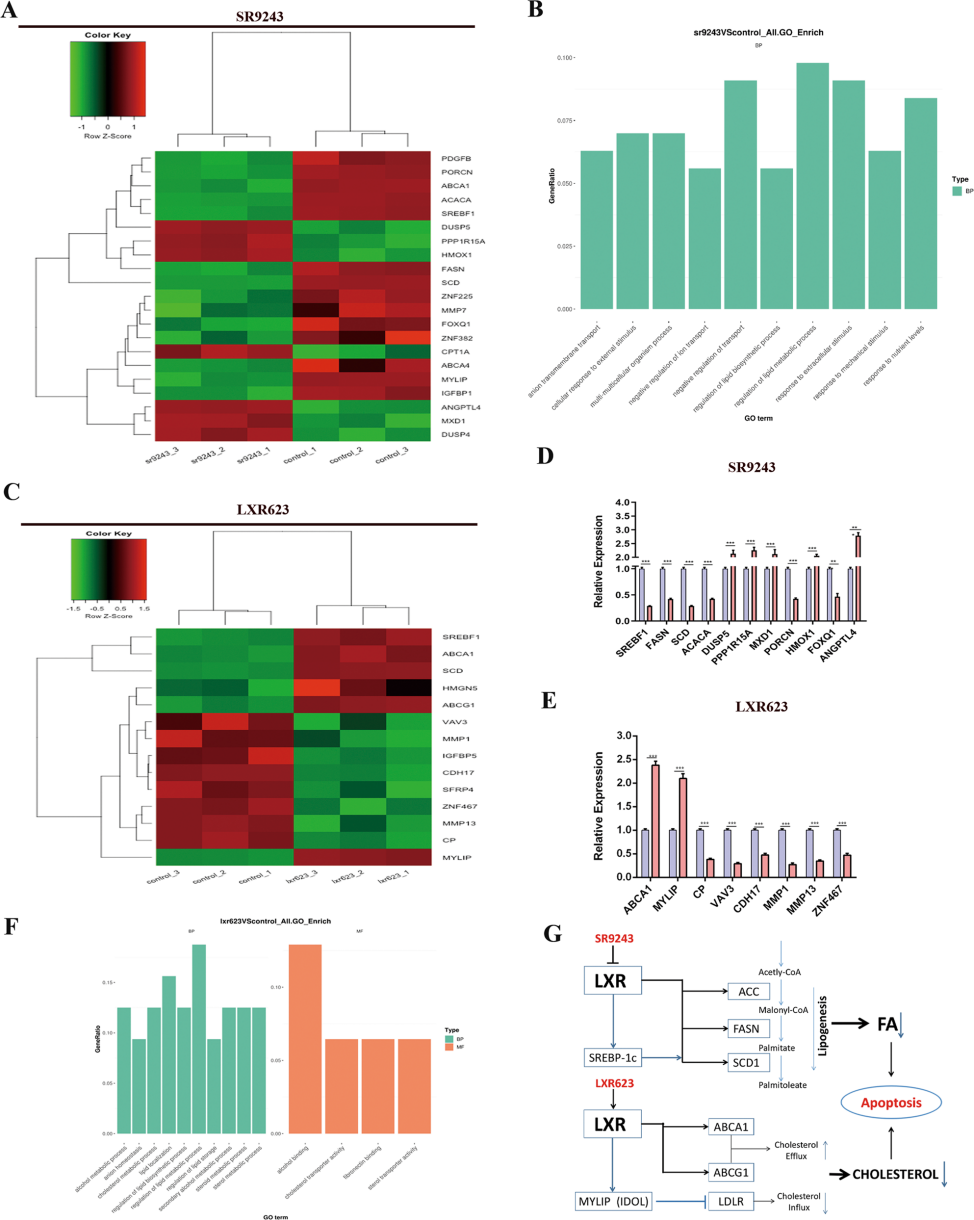
LXR623 and SR9243 inhibit ccRCC cells by inhibiting fatty acid synthesis and cholesterol transport, respectively. **a** 786-O cells were subjected to RNA-seq analysis after treatment with 0.1 µM SR9243 for 48 h. **b** Results of GO functional enrichment analysis. **c** 786-O cells were subjected to RNA-seq analysis after treatment with 0.1 µM LXR623 for 48 h. In the figure, green indicates that the gene expression is low, and red indicates that the gene expression is high. **d**, **e** RNA-seq analysis of differentially expressed genes after treatment with SR9243 and LXR623 as identified from comparison with control group expression. The data are shown as the mean ± S.E.M. Statistical analyses were performed using *t-*tests. *p < 0.05; **p < 0.01; ***p < 0.001. **f** Results of GO functional enrichment analysis. **g** Schematic showing that the LXR agonist LXR623 and inverse agonist SR9243 caused apoptosis by affecting intracellular cholesterol and fatty acid content, respectively

To study the potential underlying mechanisms, we performed RNA-seq analysis of 786-O cells treated with the two drugs. Through RNA-seq, we found that SR9243 downregulated FA synthesis genes, such as SREBP-1c, ACC, FASN and SCD1. We also found other gene changes in response to SR9243 treatment, such as a change in Porcn expression (without Porcn lipidation, WNT fails to bind to its chaperone protein)^23^. SR9243 also upregulated the PPP1R15A gene, which is involved in the TGF-β signalling pathway and promotes apoptosis by inducing the phosphorylation of TP53^24^. In addition, SR9243 upregulated the HMOX1 gene, which reduces proliferation, migration and angiogenic potential by upregulating P53^25^. SR9243 also upregulated or downregulated genes associated with tumour proliferation, apoptosis, angiogenesis, invasion and migration, such as ANGPTL4, PDGFR, DUSP5, MMP7, FOXQ1 and MXD1 (Fig. 3a, b, d)^26–31^. Different from SR9243, LXR623 upregulated the cholesterol efflux gene ABCA1 and MYLIP. MYLIP is an E3 ubiquitin ligase, which reduces LDLR protein levels by regulating ubiquitination of LDLR and, thus, its subsequent proteasomal degradation^32^. Therefore, we speculate that LXR623 kills tumour cells by promoting cholesterol efflux and inhibiting cholesterol influx. We also found other upregulated or downregulated genes related to cell proliferation, invasion and metastasis, such as VAV3, CDH17, MMP1 and MMP13^33,34^. It can be speculated that LXR agonists and inverse agonists act on tumour cells in different ways, causing decreases in intracellular FA and cholesterol contents to inhibit tumour cells (Fig. 3c, e, f). Based on the RNA-seq results, we speculate that SR9243 and LXR623 inhibit ccRCC cells by affecting intracellular FA and cholesterol content, respectively (Fig. 3g).

### SR9243 and LXR623 act on tumour cells through different lipid metabolism regulatory mechanisms

**Fig. 4:**
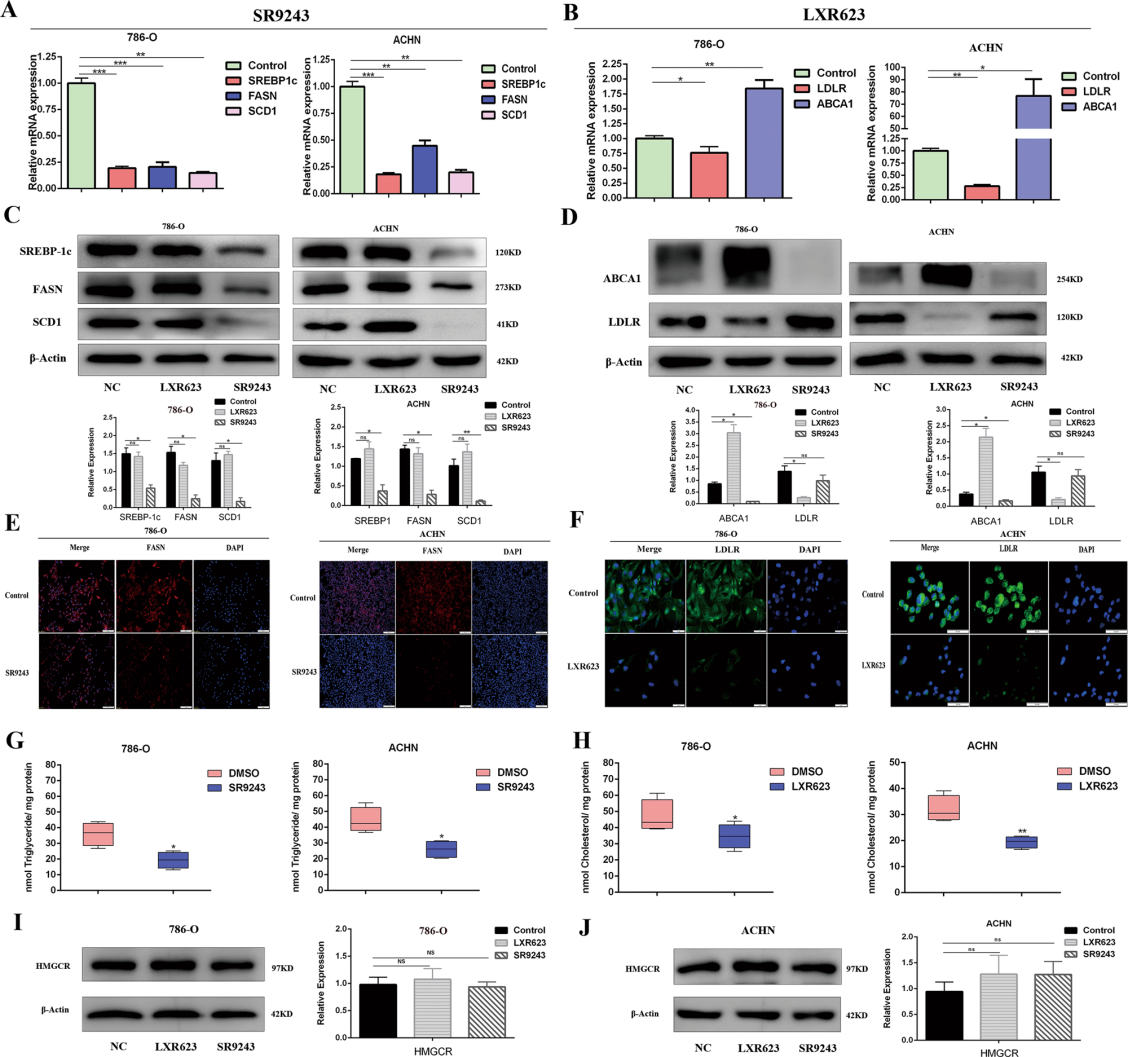
SR9243 reduces intracellular FA by inhibiting fatty acid synthesis, whereas LXR623 reduces intracellular cholesterol by affecting cholesterol transport. **a** qRT-PCR showed differences in mRNA levels of SREBP1, FASN and SCD1 48 h after treatment of cells with SR9243 (0.1 µm). **b** qRT-PCR showed differences in mRNA levels of the ABCA1 and LDLR after 48 h of cell treatment with LXR623 (786-O cells, 0.1 µM; ACHN cells, 1 µM). **c** Immunoblot assay: Three groups of cells were treated with DMSO, LXR623 (786-O cells, 0.1 µM; ACHN cells, 1 µM), or 0.1 µM SR9243 for 48 h. The protein levels of SREBP1, FASN and SCD1 were analysed via immunoblotting. **d** Immunoblot assay: Three groups of cells were treated with DMSO, LXR623 (786-O cells, 0.1 µM; ACHN cells, 1 µM), or 0.1 µM SR9243 for 48 h, and proteins were extracted. The protein levels of ABCA1 and LDLR were analysed by immunoblotting. **e** Immunofluorescence assays were performed by adding either DMSO or 0.1 µM SR9243 to cells for 48 h, and the cells were fixed and incubated with FASN antibody, followed by incubation with secondary antibody and DAPI staining. Scale bar: 200 μm. **f** Immunofluorescence: after treatment with DMSO and LXR623 (786-O cells, 0.1 µM; ACHN cells, 1 µM) for 48 h, the cells were fixed and incubated with LDLR antibody, followed by incubation with secondary antibody and DAPI staining. Scale bar: 20 μm. **g** Intracellular triglyceride assay: The intracellular triglyceride content of cells treated with SR9243 (0.1 µm) for 72 h was measured using a triglyceride kit. **h** Intracellular cholesterol assay: the intracellular cholesterol content of cells treated with LXR623 (786-O cells, 0.1 µM; ACHN cells, 1 µM) for 72 h was measured using a cholesterol kit. **i**, **j** Immunoblot assay: two cell lines were treated with DMSO, LXR623 (786-O cells, 0.1 µM; ACHN cells, 1 µM), or 0.1 µM SR9243 for 72 h, and proteins were extracted. The level of HMGCR protein was analysed via immunoblotting. All data are expressed as the mean ± S.E.M. “ns” denotes no significant difference

To confirm the RNA-seq results and further investigate the potential mechanisms by which SR9243 and LXR623 inhibit tumourigenesis in ccRCC cells, we investigated the effects of the two drugs on FA synthesis and cholesterol transport genes in the two cell lines. Through quantitative reverse transcription PCR (qRT-PCR) and western blotting (WB), we found that SR9243 significantly downregulated FASN, SREBP-1c and SCD 1mRNA and protein levels (Fig. 4a, c). The immunofluorescence experiments demonstrated that SR9243 downregulated FASN (Fig. 4e). Next, we measured intracellular triglyceride levels using a triglyceride kit. We found that SR9243 caused a decrease in intracellular triglyceride levels (Fig. 4g). We also confirmed the RNA-seq data using qRT-PCR and WB, which showed that LXR623 downregulated the cholesterol influx gene LDLR and simultaneously upregulated the cholesterol efflux gene ABCA1 (Fig. 4b, d). The immunofluorescence experiments results were consistent with the WB results (Fig. 4f). Then we used a cholesterol kit to measure intracellular cholesterol content and found that LXR623 significantly reduced intracellular cholesterol content (Fig. 4h). In addition, we performed WB to detect the expression of HMGCR, a key enzyme in cholesterol synthesis. We found that SR9243 and LXR623 did not kill tumour cells by affecting the cholesterol synthesis pathway (Fig. 4i, j).

### SR9243 and LXR623 inhibit tumour growth by reducing the intracellular FA content and cholesterol content, respectively

**Fig. 5:**
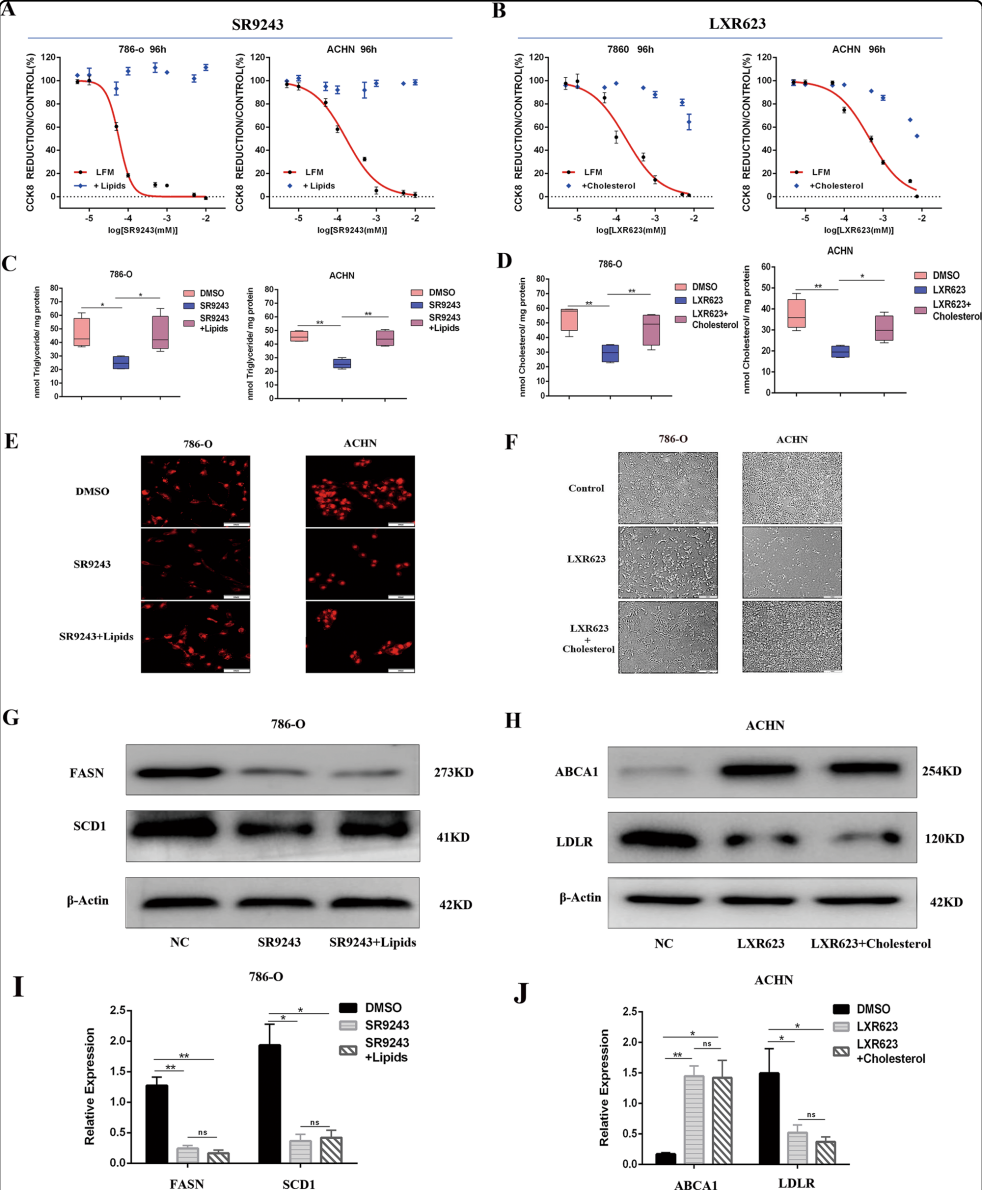
ccRCC cells are able to absorb FAs or cholesterol and rescue cell death. **a** CCK8 assay: cells cultured in LFM (fat-free medium) or positive lipid medium (addition of 25 nM oleate, palmitate and stearate) were treated with different doses of SR9243 for 96 h. **b** CCK8 assay of cells cultured in LFM (lipid-free medium) or 0.75 µg/ml cholesterol for 96 h and then treated with different doses of LXR623. **c** Triglyceride assay: The intracellular triglyceride contents in the control group, the SR9243 group and the positive lipid medium group after 72 h of treatment with SR9243 (0.1 µm) were measured using a triglyceride kit. **d** Cholesterol assay: cells were treated with LXR623 (786-O cells, 0.1 µM; ACHN cells, 1 µM) for 72 h, and the intracellular cholesterol contents in the control group, the SR9243 group, and the positive lipid medium group were measured. **e** The Nile Red assay was used to measure the intracellular lipid contents in the three groups. The three groups were the LFM group, 0.1-µM-SR9243 group and positive lipid medium group. **f** The cells in the control group, the LXR623 (786-O cells, 0.1 µM; ACHN cells, 1 µM) group and the LXR623 plus cholesterol group were observed under an inverted microscope. **g**, **i** Western blotting experiments were performed by adding DMSO, 0.1 µM SR9243, or SR9243 plus positive lipid medium (25 nM oleate, palmitate and stearate) to 786-O cells for 96 h. The protein levels of SREBP-1c, FASN and SCD1 were analysed via immunoblotting. **h**, **j** Western blotting experiments were performed by adding DMSO, 0.1 µM LXR623, or LXR623 plus 0.75 µg/ml cholesterol to 786-O cells for 96 h. The protein levels of LDLR and ABCA1 were analysed via immunoblotting

To further confirm the mechanisms by which SR9243 and LXR623 act on ccRCC cells, we designed a rescue experiment. We added FAs to the cell culture medium while applying SR9243. The addition of FAs rescued the killing effect of SR9243 and significantly increased the intracellular triglyceride content relative to that in the SR9243-only group (Fig. 5a, c). In Nile Red experiments, SR9243 significantly reduced the number of intracellular lipid droplets, but the number of lipid droplets significantly increased after exogenous FA addition. The above experiments demonstrated that ccRCC cells can take up exogenous FAs to neutralise the decline in FAs and rescue the cell death caused by SR9243 (Fig. 5e). Exogenous addition of FAs did not cause changes in the expression of proteins such as FASN and SCD1, indicating that the rescue effect of FAs was not achieved by altering the activity of LXR (Fig. 5g, i). When we added cholesterol complexed with methyl-β-cyclic amino acid (which can facilitate cholesterol import into cells) to the cell culture medium along with LXR623, we found that the exogenous cholesterol partially rescued the activity of cancer cells (Fig. 5b). The intracellular cholesterol content of the exogenous cholesterol group was increased significantly relative to that of the LXR623 group (Fig. 5d). Then, we observed the cell morphology and quantity and observed a marked rescue phenomenon after cholesterol addition (Fig. 5f). The above results confirm that LXR623 induces cell death by affecting cholesterol transport and that ccRCC cells can take up exogenous cholesterol and neutralise the decrease in cholesterol content caused by LXR623. WB showed that exogenous cholesterol did not change the expression of proteins such as LDLR and ABCA1, indicating that the rescue effect of cholesterol was not achieved by altering the activity of LXR (Fig. 5h, j).

### SR9243 and LXR623 induce ccRCC cell death without killing non-malignant cells

**Fig. 6:**
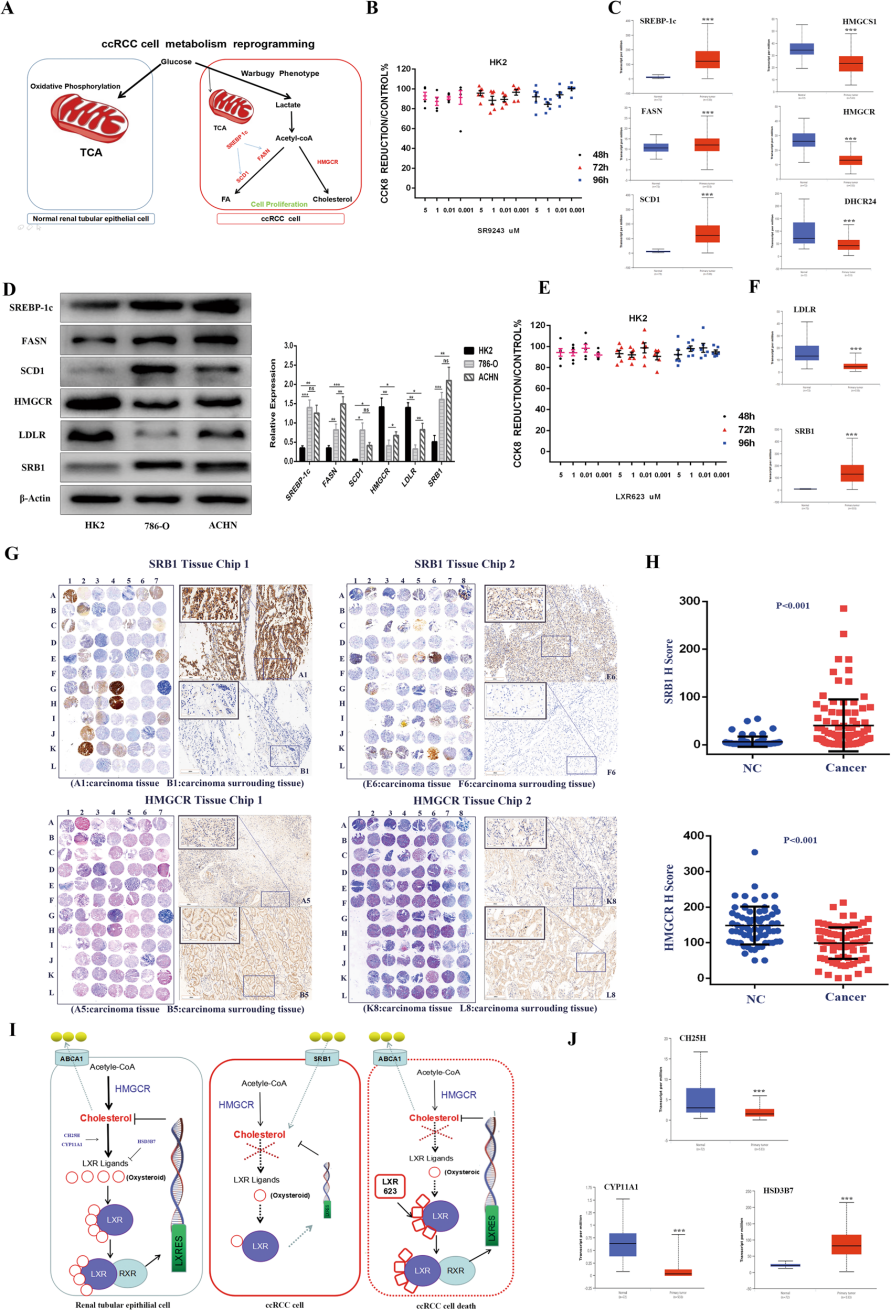
LXR623 and SR9243 have no killing effect on normal renal tubular epithelial cells. **a** Schematic diagram showing that non-malignant cells primarily support energy metabolism through the TCA; however, ccRCC cells primarily provide FAs for cell proliferation through the Warburg effect. **b** CCK8 assays were used to detect the viability of HK2 cells at different doses of SR9243 for 48, 72 and 96 h. **c** Analysis of mRNA expression differences in the SREBP-1c, FASN and SCD1 and the HMGCS1, HMGCR and DHCR24 between ccRCC and adjacent tissues in the TCGA database^65^. **d** Immunoblot assay: proteins were extracted from HK2, 786-0 and ACHN cells. The protein levels of SREBP-1c, FASN, SCD1, HMGCR, SRB1 and LDLR were analysed via immunoblotting. **e** CCK8 assays were used to detect the viability of HK2 cells at different doses of SR9243 for 48, 72 and 96 h. **f** TCGA database analysis of the cholesterol influx genes SRB1 and LDLR in ccRCC and adjacent tissues^65^. **g**, **h** HMGCR and SRB1 expression in a ccRCC tissue microarray; scale bars: 100 μm and 20 μm. The data are shown as the mean ± S.E.M. **i** Schematic showing that non-malignant cells have intact cholesterol compensatory mechanisms, whereas ccRCC cells are sensitive to exogenous LXR agonists due to a lack of endogenous LXR agonist oxysterols. **j** TCGA database analysis of the oxysterol synthesis genes CH25H and CYP11A1 and the oxysterol degradation gene HSD3B7^65^

Cancer cells primarily rely on FA synthesis to obtain the lipids needed to satisfy the demands of massive proliferation. Non-malignant cells undergo oxidative phosphate metabolism through the mitochondrial pathway and have a low level of FA synthesis ability (Fig. 6a)^35^. By performing CCK8 assays, we confirmed that SR9243 does not have a killing effect on HK2 cells (Fig. 6b). Then, through TCGA database analysis (http://ualcan.path.uab.edu), we found that SREBP-1c, FASN and SCD1 showed significantly higher expression in ccRCC specimens than in tumour-adjacent tissues (Fig. 6c). WB showed that the protein levels of SREBP-1c, FASN and SCD1 in 786-O and ACHN cells were significantly higher than those in HK2 cells (Fig. 6d). In addition, we unexpectedly found that LXR623 similarly had no killing effect on HK2 cells (Fig. 6f). LXR623 mainly affects the intracellular cholesterol content, and intracellular cholesterol is primarily obtained in two ways: self-synthesis and uptake. Therefore, we used the TCGA database to analyse the cholesterol synthesis genes and found that HMGCS1, HMGCR and DHCR24 gene expression was low in ccRCC cells (Fig. 6c), which indicated that ccRCC cells do not acquire cholesterol through synthesis. In ccRCC, the main gene for cholesterol uptake, LDLR, is also expressed at low levels. However, the high-density lipoprotein influx receptor SRB1 was highly expressed (Fig. 6f). Consistent with the TCGA database analysis, FASN, SCD1, SREBP-1c and the cholesterol influx transporter SRB1 were highly expressed in ACHN and 786O cells, whereas HMGCR and LDLR expression was low. However, FASN, HMGCR and LDLR expression was higher in the ACHN ccRCC cells than in the 7860 ccRCC cells (Fig. 6d), which suggests that ACHN cells are more dependent on the de novo synthesis of FAs and cholesterol than are 786-O cells and that intracellular cholesterol levels in 786-O cells mainly depend on SRB1 to mediate the exogenous uptake of cholesterol. In addition, based on WB and tissue microarray (TMA) data, we found that HMGCR expression was lower and SRB1 expression was higher in tumours than in tumour-adjacent tissues (Fig. 6d, g). These results indicated that non-malignant cells primarily acquire cholesterol through synthesis, whereas tumour cells primarily obtain cholesterol through uptake by SRB1. As LXR is primarily activated by LXR ligand and because oxysterol is the main endogenous LXR ligand in cells, we used the TCGA database to analyse the synthesis and decomposition genes of the endogenous LXR ligand (oxysterol) in ccRCC and adjacent tissues. We found that in ccRCC, the endogenous ligand synthesis genes CYP11A and CH25H were expressed at low levels and that the decomposition (oxysterol) enzyme HSD3B7 had a nearly 10-fold higher expression in ccRCC than in adjacent tissue (Fig. 6d, i). These results showed that the ccRCC tissue had a reduced ability to produce oxysterols and an increased ability to decompose oxysterols. Therefore, the ccRCC cells were in a state of oxysterol (LXR endogenous ligand) deficiency. This lack of endogenous agonists indicates that exogenous LXR agonists (LXR623) have a strong stimulatory effect on ccRCC cells but do not have a lethal effect on HK2 cells (Fig. 6h). ACHN cells are more dependent on cholesterol synthesis than are 786-O cells, and thus, LXR623 had a weaker killing effect on ACHN cells than on 786-O cells (Fig. 1b, d).

### Antitumour effect of SR9243 in vivo

**Fig. 7:**
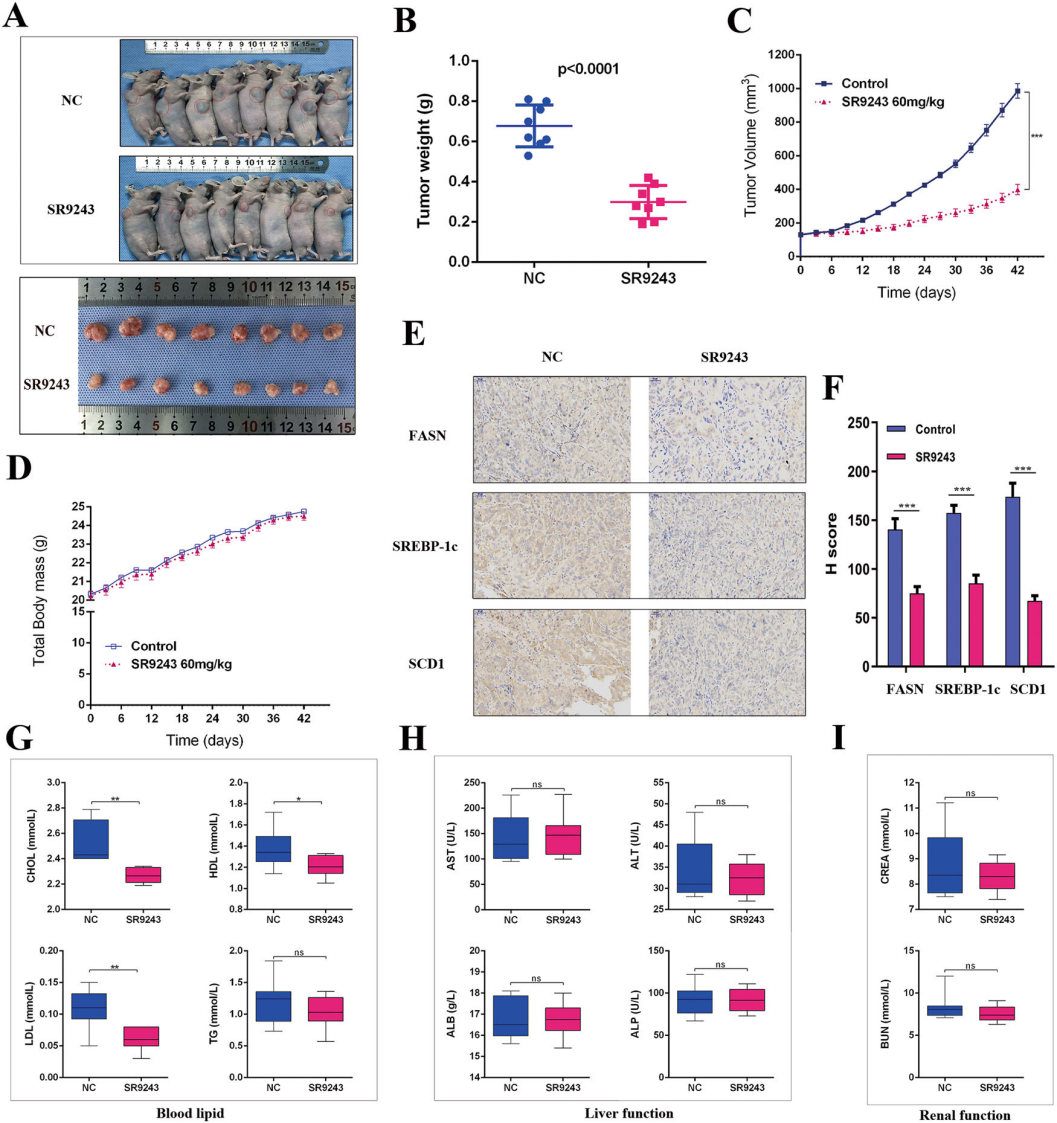
Antitumour effect of SR9243 in vivo. **a** Nude mice were treated with normal saline (*n* = 8) or 60 mg/kg (*n* = 8) SR9243 via intraperitoneal injection for 42 days, and photographs of the tumours were obtained at autopsy. **b** Scatter plot analysis of tumour weights in nude mice. **c** Tumour volume was measured every 3 days. All data are expressed as the mean ± S.E.M. **d** The body weight of nude mice was measured every 3 days. The image shows the effect of SR9243 on the body weight of tumour-bearing nude mice. **e** IHC was used to detect differences in FASN, SCD1 and SREBP-1c expression between the two tumour groups. Scale bar: 50 µm. **f** IHC staining score for each protein (**e**) in the two groups. The data are expressed as the mean ± S.E.M. **g** CHOL, TRIG, LDL and HDL levels in the two groups. **h**, **i** Plasma levels of ALP, AST, ALB, ALT, CREA and BUN in the two groups

To investigate the role of SR9243 in the growth of ccRCC cells in vivo, we established a nude mouse xenograft model. Photographs of tumour tissue and tumour volume indicated that SR9243-treated 786-O cell xenografts grew much more slowly than control group (Fig. 7a, c). The weight of tumours from the SR9243-treated mice was significantly lower than that of tumours from control mice (Fig. 7b). Immunohistochemistry analysis showed that the expression of FASN, SCD1, and SREBP-1c in the SR9243 group was significantly lower than that in the control group (Fig. 7e, f). FA synthesis inhibitors have been reported to produce side effects, such as weight loss and loss of appetite^36,37^. However, unlike nude mice treated with FA synthesis inhibitors, the nude mice treated with the LXR inverse agonist SR9243 did not show any signs of weight loss (Fig. 7d). Since LXR agonists have been shown to increase cholesterol reverse transport by increasing the expression of extrahepatic ABCA1^38–40^, we were concerned that SR9243 may reduce the expression of extracellular ABCA1 and thus reduce the reverse transport of cholesterol, leading to an increase in plasma cholesterol. Related studies have found that LXR inverse agonists are rapidly metabolised in the liver, providing extensive liver exposure rather than peripheral plasma exposure. Therefore, we performed a biochemical examination of plasma from the nude mice and found that SR9243 reduced plasma TC, LDL and HDL levels and had no significant effect on triglycerides (Fig. 7g). SR9243 reduced blood lipids but had no significant effect on liver and kidney function (Fig. 7h, i), which is consistent with previous reports^7,41^. In summary, SR9243 inhibits lipogenesis and induces apoptosis in ccRCC cells without hepatotoxicity or nephrotoxicity.

## Discussion

**Fig. 8:**
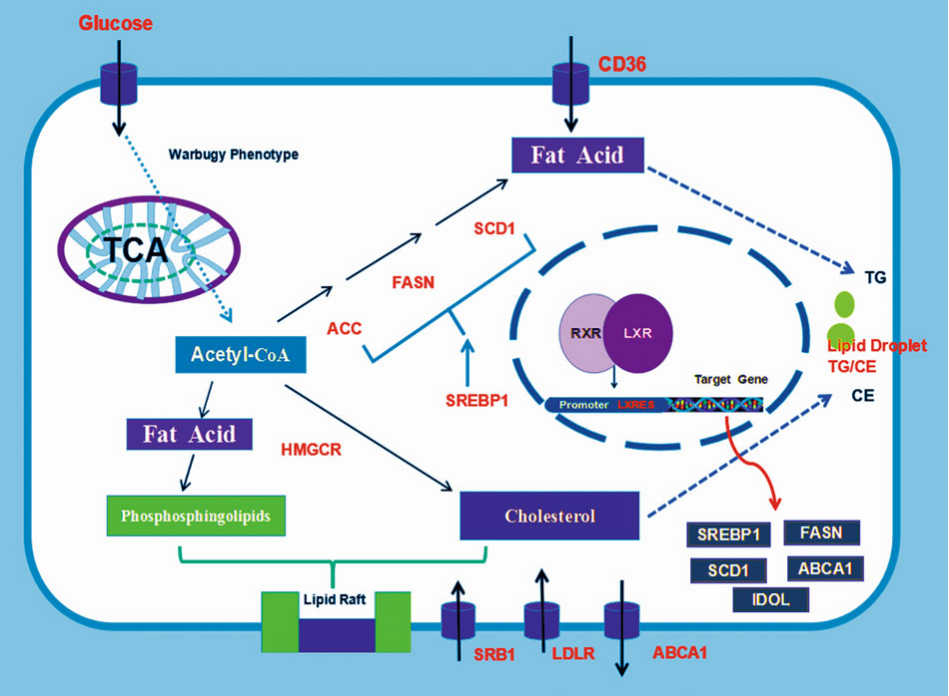
**Mechanism of LXR regulation of lipid metabolism**

Tumour cells often utilise aerobic glycolysis (the Warburg effect) to meet energy and membrane structure requirements. This is a key metabolic pathway that drives cancer progression, growth, survival, immune evasion and disease recurrence and is more pronounced in ccRCC than in healthy tissues^6^. The glycolysis products are used in lipid de novo synthesis, providing substances for cell proliferation; thus, targeting lipid metabolism may play a significant role in ccRCC^6,42,43^. The nuclear receptor LXR can directly regulate the expression of various genes in lipogenesis, and the LXR inverse agonist SR9243 inhibits the transcription of target genes by inducing interactions between LXR and co-repressors. However, the effect of SR9243 in ccRCC has not been reported previously. Here, we experimentally demonstrated that the LXR inverse agonist SR9243 can significantly inhibit the proliferation of 7860 and ACHN cells and induce apoptosis through the endogenous apoptotic pathway. We found that SR9243 downregulated the lipogenesis genes SREBP-1c, FASN and SCD1, which reduced the intracellular FA content to exert a tumour suppressor effect, and that ccRCC cells could exogenously take up FAs to rescue tumour death. In addition, the LXR agonist LXR623 had a pronounced killing effect on ccRCC cells. LXR623 significantly reduced the intracellular cholesterol content by upregulating ABCA1 and downregulating LDLR and induced ccRCC cell apoptosis. Exogenous cholesterol intake can rescue cell death. As both LXR agonists and inverse agonists cause cell death, we hypothesise that LXR plays a crucial regulatory role in the balance between FAs and cholesterol. A certain level of LXR expression maintains intracellular FAs and cholesterol in a balanced state that can satisfy the proliferation requirements of tumour cells. An LXR agonist or reverse agonist can destroy this balance, thereby reducing intracellular FA or cholesterol levels and inhibiting tumours (Fig. 8). This hypothesis requires experiments for verification.

Lipid rafts are composed of cholesterol and phospholipids. Studies have shown that rate-limiting enzymes in FA synthesis affect the phospholipid composition of cell membranes^44–47^. The distribution of FAs and cholesterol in lipid rafts is highly dynamic and can serve as a selective signal transduction pathway that mediates lipid metabolism, cell survival, adhesion, metastasis, and tumour progression^48–52^. Inhibition of the LXR target gene FASN can affect the lipid synthesis associated with lipid rafts^53^. Changes in membrane cholesterol levels have been shown to affect cancer invasiveness and progression^54,55^. The FAs and cholesterol in cellular lipid metabolism are involved in a network of pathways exhibiting flexibility, feedback loops and crosstalk, which are regulated to meet the growing metabolic needs of cancer cells^56^. Therefore, by causing FA or cholesterol reductions, an LXR agonist or inverse agonist might affect cell function, causing membrane structural function disruption, cell signal transduction pathway disorder or cell death. Since the proliferation of tumour cells requires high levels of FAs and cholesterol in cells, we investigated the expression of FA synthesis genes and cholesterol synthesis transporter proteins in different experiments. We found that ccRCC cells had higher expression of the FA synthesis genes SREBP-1c, FASN and SCD1 than did HK2 cells, which is consistent with our hypothesis that the increase in FAs is important because they are components of membrane structures required for ccRCC cell proliferation. However, the expression of the cholesterol synthesis genes DHCR24, HMGCS1 and HMGCR was lower in ccRCC cells than in adjacent tissues, and thus, we concluded that the cause of the higher cholesterol content in ccRCC is not cholesterol synthesis but exogenous uptake. We then analysed cholesterol influx and efflux proteins and found that in ccRCC cells, LDLR expression was low, whereas SRB1 expression in ccRCC cells and tissue was several hundred times higher than that in adjacent cells. These findings indicated that the high cholesterol level in ccRCC may be partly related to SRB1-mediated HDL uptake.

To effectively destroy tumour metabolism, SR9243, an enzyme-specific inhibitor drug, can be used. SR9243 selectively targets and destroys metabolic processes in tumour cells with high metabolic activity while not harming non-malignant cells that have lower metabolic activity^8^. It is well known that while non-malignant cells primarily utilise glucose metabolism through the TCA cycle, tumour cells employ aerobic glycolysis to utilise glucose. Metabolites, such as lactic acid, produced via aerobic glycolysis can provide large numbers of membrane structures for tumour growth and energy. Non-malignant cells are primarily characterised by low levels of FA synthesis, and for most lipogenic genes, such as ACLY and FASN, there is no “backup” gene product to replace the function of the targeting molecule. Therefore, inhibiting the lipogenesis pathway may not have a lethal effect on non-malignant cells^57–59^. SR9243 can produce a metabolic environment that does not support the growth of cancer cells but has no effect on the function of non-malignant cells. Therefore, SR9243 may be a safe drug for the clinical treatment of ccRCC.

Another unexpected finding of the present study is that LXR623 can also induce ccRCC cell death without killing HK2 cells. The reason for this ability may be related to the following points: First, oxysterol is a metabolite of cholesterol and an endogenous agonist of LXR, and cholesterol synthesis inhibitors may reduce the content of oxysterols in cells, thereby weakening the reactivity with LXR^60,61^. Oxysterol-synthesising knockout mice, such as 24S-hydroxycholesterol and 25-hydroxycholesterol knockout mice, have been shown to induce certain LXR target genes in the liver when fed cholesterol, indicating that LXR activates different types of target genes depending on the ligand and that part of the endogenous LXR ligand (oxysterol) is derived from decomposition of the cholesterol produced by de novo synthesis^62^. The cholesterol synthesis gene is expressed at low levels in ccRCC cells, and thus, ccRCC cells may have lower oxysterol synthesis ability. Second, non-malignant cells were not sensitive to LXR623, which may be due to a dependence on endogenous cholesterol synthesis and complete negative feedback via the synthesis of endogenous oxysterols. For example, when cholesterol is sufficient, normal cells convert excess cholesterol to oxysterol to stimulate LXR, promote cholesterol efflux, and reduce intracellular cholesterol content; however, ccRCC cells behave differently. ccRCC cells exhibit low expression of CH25H and CYP11A1 genes, which are enzymes that convert cholesterol to oxysterols. Furthermore, TCGA data indicate that the enzyme HSD3B7, which decomposes the LXR ligand (oxysterols), exhibits nearly 100-fold higher expression in ccRCC cells than in normal cells, which explains the endogenous LXR ligand deficiency in ccRCC. The lack of endogenous LXR ligands leads to ccRCC sensitivity to the exogenous LXR agonist LXR623; however, non-malignant cells have a complete negative feedback mechanism, and therefore, LXR623 does not kill HK2 cells. In addition, the scarcity of endogenous ligands in ccRCC cells prevents reduction of the intracellular cholesterol content through the LXR agonistic pathway, which results in higher cholesterol accumulation in ccRCC cells and in part explains the origin of “kidney clear cell carcinoma” (the high levels of cholesterol and cholesterol esters in kidney cancer tissue specimens).

In our experiments, we found a large difference in the killing effect of LXR623 between the 786-O and ACHN cell lines. The killing of ACHN cells required a larger dose of LXR623. 786-O cells have many primary ccRCC characteristics, whereas ACHN cells are metastatic cells isolated from pleural effusion metastasis^63^. The expression of HMGCR in ACHN cells was significantly higher than that in 786-O cells, indicating that ACHN cells are more dependent on HMGCR-mediated cholesterol synthesis than are 786-O cells; accordingly, LXR623 had a weaker killing effect on ACHN cells than 786-O cells. Currently, molecular targeted therapy is mainly applied in mRCC, but LXR623 had a poor killing effect on mRCC ACHN cells. In contrast, SR9243 killed tumour cells under the action of a small IC50 in both cell lines. Thus, we believe that SR9243 would be more effective than LXR623 in the clinical treatment of ccRCC. Therefore, in this study, we conducted in vivo experiments with only SR9243.

To obtain lipids, cancer cells are more dependent on lipid synthesis than on exogenous sources. Increased expression of lipogenic enzymes is the basis of the metabolic changes, and thus, targeting FA synthesis is a new direction in cancer therapy. However, the use of inhibitors against these enzymes has been restricted due to their adverse effects, such as severe weight loss and hepatotoxicity^35^. In our experiments, we found that SR9243 targets a variety of key enzymes in FA production and that SR9243 significantly inhibited the growth of subcutaneous xenografts in nude mice but had no effect on the weight or the liver and kidney functions of nude mice. Overall, SR9243 may be a safe and effective drug for treating ccRCC.

## Materials and methods

### Cell culture, antibodies and reagents

The 786-O, ACHN and HK2 were purchased from the Chinese Academy of Sciences cell bank. All cells were cultured in the presence of penicillin/streptomycin at 37 °C in air containing 5% CO_2_. The antibodies used included mouse anti-ABCA1 (Abcam, ab18180), rabbit anti-LDLR (Abcam, ab30532), rabbit anti-HMGCR (Abcam, ab174830), rabbit anti-SREBP-1c (Abcam, ab28481), mouse anti-SCD1 (Abcam, ab19862), rabbit anti-SRB1 (Abcam, ab217318), rabbit anti-FASN (CST, 3180), rabbit anti-Bax (Abcam, ab32503), rabbit anti-Bcl2 (Abcam, ab59348), rabbit anti-Caspase3 (Abcam, ab32042), and anti-HMGCR (used for IHC, Proteintech, 13533). Cholesterol-methyl-β-cyclodextrin (Sigma, C4951), SR9243 (MCE, 16972), LXR623 (MCE 10629), stearic acid (MCE, B2219), oleic acid (MCE, N1446) and palmitic acid (MCE, N0830) were also utilised.

### RNA-seq

Total RNA was extracted from cells with Trizol reagent (Invitrogen, CA, USA). Random hexamer-primers were used to synthesise first-strand cDNA. Second-strand cDNA was synthesised using buffer, dNTPs, RNase H and DNA polymerase I. After adapter ligation and agarose gel electrophoresis, suitable fragments were selected for PCR amplification. Finally, the RNA-seq library was sequenced using an Illumina Novaseq 6000 PE150 instrument by Haplox Biotechnology Co. (ShenZhen, China).

### Tissue microarray technology

From February 2012 to June 2015, cancer tissues and their corresponding adjacent normal tissues from 90 cases were obtained from patients who underwent ccRCC surgery at Shandong Provincial Hospital. For each patient, both the patient and a family member signed an informed consent agreement. All 180 tissues were used to create tissue microarrays (manufactured by Wuhan Servicebio Technology Co, Ltd). The tissue microarrays were stained using a semi-quantitative system according to the manufacturer’s instructions and verified by immunohistochemistry. Protein staining was carried out with DAB enzyme (Abcam, ab64238), and the nuclei were stained with haematoxylin (Abcam, ab143166).

### Immunofluorescence

Cells were fixed with 3% paraformaldehyde and permeabilized with 0.1% Triton X-100. The cells were then incubated with the appropriate primary antibody and fluorescently labelled secondary antibody. The Alexa Fluor 488- and 594-conjugated secondary antibodies were purchased from Invitrogen (CA, USA). DNA was stained with DAPI (Abcam, ab104139), and immunostained cells were photographed using an inverted fluorescence microscope (Thermo Fisher, USA).

### RNA extraction and real-time PCR

Total RNA was extracted with Trizol reagent (Takara, 9108). cDNA was synthesised from 2 μg RNA using the Quantscript RT Kit (Takara, PR047A) according to the manufacturer’s instructions. The primer sequences used for real-time PCR were as follows: ABCA1-F, 5′-GGATCATGGGCCTGGACAA-3′; ABCA1-R, 5′-GGGATCACTGTAGGGCAGCA-3′; FASN-F, 5′-AGCACAGACGAGAGCACCTTTG-3′; FASN-R, 5′-CCATGCAGCTCAGCAGGTCTA-3′; LDLR-F, 5′-CTGGTCAGATGAACCCATCAAAGA-3′; LDLR-R, 5′-TCATTGCAGACGTGGGAACAG-3′; SREBF1-F, 5′-CCTAAGTCTGCGCACTGCTGTC-3′; SREBF1-R, 5′-CCATGAGCACGTCTGTGTTCC; SCD1-F, 5′-GCTACACTTGGGAGCCCTGTATG-3′; SCD1-R, 5′-AGACGATGAGCTCCTGCTGTTATG-3′; HA067803-F, 5′-TGGCACCCAGCACAATGAA-3′; and HA067803-R, 5′-CTAAGTCATAGTCCGCCTAGAAGCA-3′.

### Clone formation assay

After the drug was applied to cells for a certain period of time, the cells were inoculated into a new culture dish. The cells were uniformly dispersed and cultured in a cell culture incubator for 2 weeks. When macroscopic clones appeared in the culture dish, the culture was terminated and washed twice with PBS. The clones were stained with crystal violet (MCE, B0324A), and rinsed with double distilled water. Then, the dish was inverted for counting.

### Flow cytometry

The cell suspension was washed twice with precooled PBS. Then, 500 mg binding buffer was added to each tube and transferred to a centrifuge tube (1 to 5 × 10^5^ cells). Next, 5 μl Annexin-V-FITC and 5 μl propyl propionate (BD, 559763) were added. The cells were incubated for 15 min at room temperature in the dark and analysed via flow cytometry (BD, NJ, USA). At least 10,000 cells were collected. The results were analysed using FlowJo 7.6.2 software (BD, USA).

### Cell counting kit 8 assay

Cell proliferation ability was evaluated using Cell Counting Kit-8 (Dojindo, Japan). Appropriate cells were seeded in 96-well plates, and after a specified period of drug treatment, 10 µl of CCK8 was added to the corresponding wells and incubated with cells for 30 min in a 5% CO_2_ incubator. The optical density (OD) of each well was measured with a microplate reader at a wavelength of 450 nm.

### Ethynyl-2′-deoxyuridine (EdU) assay

Cells were seeded in 24-well plates and incubated with medium supplemented with 50 µM EdU for 3 h. Then, the cells were fixed with 4% paraformaldehyde for 30 min at room temperature, permeabilized for 15 min in 0.5% Triton X-100, and stained with Hoechst for 30 min at room temperature. Finally, images were obtained with a microscope (Olympus, Tokyo, Japan) and analysed using Image-Pro Plus software (Media Cybernetics, Bethesda, MD, USA).

### Western blotting

Protein was extracted from cells using RIPA lysis buffer (CST, 9806), and protein concentration was determined using the bicinchoninic acid (BCA) method (Abcam, ab102536). Each sample (30 µg protein) was separated on a 6–15% sodium dodecyl sulfate polyacrylamide gel electrophoresis gel (Keygen Biotech, KGP113K), transferred to a polyvinylidene fluoride (PVDF) membrane (Millipore, R8CA8257B), blocked with 5% skim milk powder and incubated with primary antibody overnight. The membrane was then incubated with peroxidase-conjugated secondary antibody, and the immunoreactive bands were observed using an ECL system.

### Nile red assay

Nile Red (MCE, D0718) was used for neutral lipid staining. Cells were incubated with 1 µM Nile Red solution in a 37 °C incubator for 10 min and observed under a fluorescence microscope at EX/EM = 530/635 nm.

### Determination of intracellular triglyceride and cholesterol contents

Intracellular triglyceride and cholesterol contents were determined using a commercial kit (Applygen Technologies, Beijing, China) according to the manufacturer’s protocol^64^.

### Xenografts

A suspension of 786-O cells containing 5 × 10^6^ cells was injected subcutaneously into the right axilla of nude mice (SPF grade, 4-weeks-old, eight per group). SR9243 or saline was injected into the mice when the tumour volume in each nude mouse was greater than 100 mm^3^. After 42 days of treatment, the mice were sacrificed, and the tumours were resected for evaluation.

### Statistics

The data are expressed as the mean ± S.E.M. Statistical analysis was performed using Prismsoftware (GraphPad, CA, USA). Statistical significance of differences between and among groups was assessed using *t*-test and one-way ANOVA, respectively. Significant differences are indicated as follows: *p < 0.05; **p < 0.01; ***p < 0.001.
